# Altered Cerebral Blood Flow as a Screening Test for Neurodegenerative Disease: A Comparative Analysis of Arterial Spin Labeled Magnetic Resonance Imaging and 18F‐Fluorodeoxyglucose Positron Emission Tomography

**DOI:** 10.1002/alz.092331

**Published:** 2025-01-09

**Authors:** Andres Ricaurte Fajardo, Ilhami Kovanlikaya, Weiye Yasen, Meem Mahmud, Jana Ivanidze, Yi Li, Mony J. de Leon, Tracy A Butler, Anna S Nordvig, Gloria Chiang

**Affiliations:** ^1^ Weill Cornell Medicine, New York City, NY USA; ^2^ Brain Health Imaging Institute, Department of Radiology, Weill Cornell Medicine, New York City, NY USA

## Abstract

**Background:**

Early detection of Alzheimer’s disease (AD) can improve prognosis, given new anti‐amyloid therapies. Both positron emission tomography (PET) and magnetic resonance (MR) imaging biomarkers are currently used (1). 48F‐Fluorodeoxyglucose‐PET (FDG‐PET) can detect neurodegeneration‐related hypometabolism but is costly and not easily accessible (2). Cerebral blood flow (CBF), assessed using arterial spin labelling magnetic resonance imaging (ASL‐MRI), is closely linked to metabolism in neurodegenerative disease (2). In a recent case series of seven neurodegenerative patients, we described concordance between CBF and (i) FDG‐PET and (ii) amyloid/tau PET (3). In this clinical cohort, ASL‐MRI was used to triage patients with cognitive concerns.

**Method:**

A retrospective analysis included patients aged 25‐85 with cognitive symptoms, who underwent ASL‐MRI and either (i) FDG‐PET (n = 65) and/or (ii) amyloid PET or CSF sampling for amyloid/tau (n = 44). CBF maps were standardized and independently assessed by two neuroradiologists for a typical AD pattern (temporoparietal) or lobar (≥2 lobes) hypoperfusion. FDG avidity was compared against a normative database for regional hypometabolism. The sensitivity and specificity of ASL‐MR in identifying AD was assessed, as well as discordance with FDG‐PET.

**Result:**

82 patients met inclusion criteria (mean age 68±13), interrater reliability for lobar hypoperfusion was 84%. Temporoparietal hypoperfusion was 81% specific, but only 52% sensitive for biomarker‐proven AD; lobar hypoperfusion increased sensitivity to 78%, but specificity decreased to 43%. Using temporoparietal hypoperfusion, there was 29% discordance with FDG‐PET. Using lobar hypoperfusion, there was 38% discordance.

**Conclusion:**

ASL‐MRI is a promising, non‐invasive screening tool for early detection and diagnosis of AD and other neurodegenerative diseases. Understanding the discordance between ASL‐MRI and FDG‐PET warrants further attention. In routine assessments for cognitive complaints, ASL‐MRI should be added to the screening protocol MRI.

**Bibliography**:

1. Haidar H, Majzoub RE, Hajeer S, Abbas LA. Arterial spin labeling (ASL‐MRI) versus fluorodeoxyglucose‐PET (FDG‐PET) in diagnosing dementia: a systematic review and meta‐analysis. BMC Neurol. 2023 Oct 24;23(1):385.

2. Minoshima S, Cross D, Thientunyakit T, Foster NL, Drzezga A. ^18^F‐FDG PET Imaging in Neurodegenerative Dementing Disorders: Insights into Subtype Classification, Emerging Disease Categories, and Mixed Dementia with Copathologies. J Nucl Med. 2022 Jun;63(Suppl 1):2S‐12S.

3. Accepted publication, AJNR.

